# Transcriptional changes impact hepatic proteome in autophagy‐impaired liver

**DOI:** 10.1002/2211-5463.13898

**Published:** 2024-09-16

**Authors:** Kamal Baral, Spandan Joshi, Adriana Lopez, Gavisha Mugon, Aroma Chanda, Arya A. Chandrasheker, Cameron Hinton, Kapil Thapa, Arissa Mercer, Leah Spade, Gang Liu, Bhupal Prasad Bhetwal, Jia Fang, Bilon Khambu

**Affiliations:** ^1^ Department of Pathology and Laboratory Medicine Tulane University School of Medicine New Orleans LA USA; ^2^ Wabash College Crawfordsville IN USA; ^3^ Department of Biochemistry and Molecular Biology Tulane University School of Medicine New Orleans LA USA; ^4^ Department of Cell and Molecular Biology School of Science and Engineering New Orleans LA USA; ^5^ Hackensack Meridian School of Medicine Nutley NJ USA

**Keywords:** autophagy, hepatic proteome, liver, non‐degradative, Nrf2, transcriptional repression

## Abstract

Hepatic proteomes are intricately controlled through biosynthesis, extracellular secretion, and intrahepatic degradation. Autophagy governs lysosome‐mediated intrahepatic degradation and the hepatic proteome. When autophagy is impaired, it leads to the accumulation of intrahepatic proteins, causing proteinopathy. This study investigates whether autophagy can modulate the hepatic proteome non‐degradatively. Utilizing conditional, inducible, and hepatotoxin models of hepatic autophagy impairment, we assessed the overall hepatic proteome expression using Coomassie brilliant blue (CBB) staining and liquid chromatography–tandem mass spectrometry (LC/MS). We pinpointed and confirmed four specific hepatic proteins—Cps1, Ahcy, Ca3, and Gstm1—that were selectively modified in autophagy‐deficient livers. Expression of Cps1, Ahcy, and Ca3 were significantly reduced, while Gstm1 expression increased in livers with autophagy impairment. Interestingly, these changes in hepatic protein levels were not due to defective autophagic degradation but were associated with alterations in mRNA transcript levels. Moreover, as a result of autophagic dysfunction, sustained activation of the nuclear erythroid‐derived 2‐like 2 (Nrf2) transcription factor, transcriptionally regulated the mRNA levels of these proteins. Our findings indicate that autophagy can influence hepatic proteins not solely via traditional degradative routes but also through non‐degradative transcriptional processes by modulating Nrf2.

## Graphical abstract

Autophagy can regulate the hepatic proteome through the transcriptional non‐degradative pathway in addition to the conventional lysosomal degradative pathway.

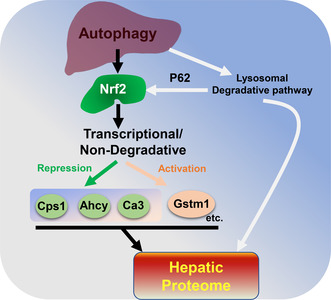

**Abbreviations**
4‐OHT: tamoxifen
Ahcy: adenosyl homocysteinase
ARE: antioxidant response element
Atg5: autophagy related protein 5
Atg7: autophagy related protein 7
BCA: bicinchoninic acid
Ca3: carbonic anhydrase 3
CBB: Coomassie brilliant blue
Cps1: carbamoyl phosphate synthetase I
Cry1: cryptochrome circadian regulator 1
DDC: 3,5‐diethoxycarbonyl‐1,4‐dihydrocollidine
FXR: farnesoid X receptor
Gstm1: glutathione *S*‐transferase mu 1
Keap1: Kelch‐like ECH‐associated protein 1
LC/MS: liquid chromatography tandem mass spectrometry
MafG: musculoaponeurotic fibrosarcoma transcription factor G
NcoR1: nuclear receptor corerepressor 1
Nrf2: nuclear factor erytheroid‐derived‐2‐like 2
NTum: non‐tumor
P62/SQSTM1: sequestosome‐1
PSM: peptide spectrum match
RPA1: replication protein A 1
SAH: *S*‐adenosylhomocysteine
Tum: tumor
Yap: yes‐associated protein 1

The liver synthesizes and degrades both intracellular and secretory proteins. There is an ongoing process of synthesis and degradation of intracellular protein to regulate the cytoplasmic mass of the hepatic proteome. Disbalance in hepatic protein degradation or synthesis results in intrahepatic protein accumulation such as Mallory Dek Bodies to cause proteinopathy [1]. Hepatic proteomes are short‐lived (having a half‐life of approximately 10 min and comprising about 0.6% of total hepatic protein) or long‐lived (with an average half‐life of 250 times greater, constituting more than 99% of the hepatic proteins) [2].

Macroautophagy hereafter termed autophagy is an evolutionarily conserved intracellular lysosome‐mediated degradative pathway that can selectively or non‐selectively target various intrahepatic proteins [3]. Autophagic non‐selective protein degradation involves sequestration of cytosolic protein aggregates by an expanding membrane called a phagophore which then forms autophagosomes that can later fuse with lysosomes to form autolysosomes for degradation and recycling. Selective autophagic protein degradation is mediated by autophagy receptors such as P62/SQSTM1 [4, 5]. These autophagy receptors link protein cargoes and nascent autophagosomes through the LC3‐interacting region [5]. Protein cargoes are ubiquitinated and recognized by autophagy receptors with a ubiquitin‐binding domain and eventually transported to the lysosome for degradation. Impaired hepatic autophagy results in the accumulation of multiple proteins such as P62/SQSTM1 [6]. Thus, autophagy is a central regulator of the hepatic proteome.

Autophagy is well known for modulating the hepatic proteome through lysosome‐mediated intracellular degradation. It remains unclear if autophagy can also regulate the hepatic proteome non‐degradatively. This study demonstrates that autophagy can affect hepatic proteome through non‐degradative transcriptional modulation via nuclear factor erytheroid‐derived‐2‐like 2 (Nrf2). For instance, compromised autophagy can selectively modify the abundance of hepatic proteins, such as Cps1, Ahcy, Ca3, and Gstm1.

## Results

### Hepatic proteome is qualitatively altered in conditional autophagy‐deficient liver

In the liver, protein homeostasis is regulated by autophagy. Deletion of autophagy function in liver can directly impact the proteome degradation and alter the qualitative or quantitative proteome profile. To examine the hepatic proteomic profile in absence of autophagy function, liver‐specific Atg7 knock‐out mouse was created by crossing Atg7 floxed mice (Atg7F/F) with Alb‐Cre mice (Fig. S1A). Atg7 is an autophagy gene encoding E1‐like enzyme in the two ubiquitin‐like conjugation systems that are essential for the autophagosome biogenesis [7]. Deletion of Atg7 blocks autophagosome formation and hence inhibits autophagy function. Western blot and immunofluorescence analysis of Atg7, P62, and LC3B confirmed the liver‐specific knockout of Atg7 (Fig. S1B,C).

**Fig. 1:**
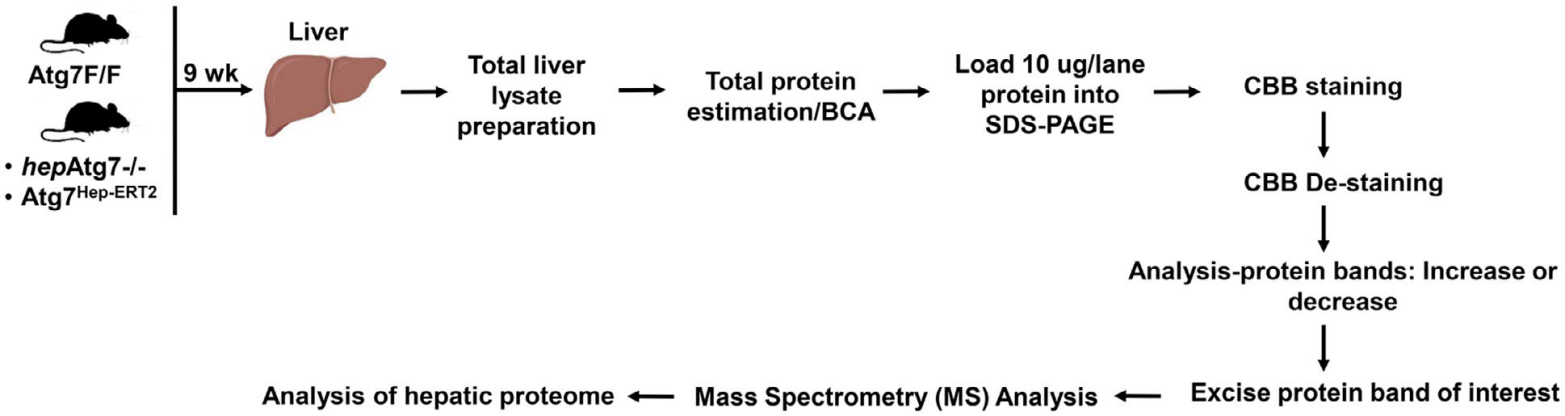
Experimental outline for examining hepatic proteome in autophagy‐deficient liver. Liver lysates from 9‐weeks old wild‐type, Atg7F/F and hepAtg7−/− or Atg7^Hep‐ERT2^ mice were prepared and estimated for total protein amount by bicinchoninic acid (BCA) protein assay. SDS/PAGE was performed by loading protein (10 μg/lane). Gels were stained for Coomassie brilliant blue (CBB) to analyze protein bands between the experimental groups. Furthermore, specific protein bands were excised and analyzed by mass spectrometry (MS) to identify the specific hepatic proteome.

To determine the hepatic proteome in normal and autophagy‐deficient liver, 10 μg of total liver lysate was loaded into the SDS/PAGE and stained with Coomassie brilliant blue staining (CBB) stain. Alteration of overall total proteins band and specific protein bands were examined. Specific altered protein bands were then excised and examined by LC/MS (Fig. 1).

**Fig. 2:**
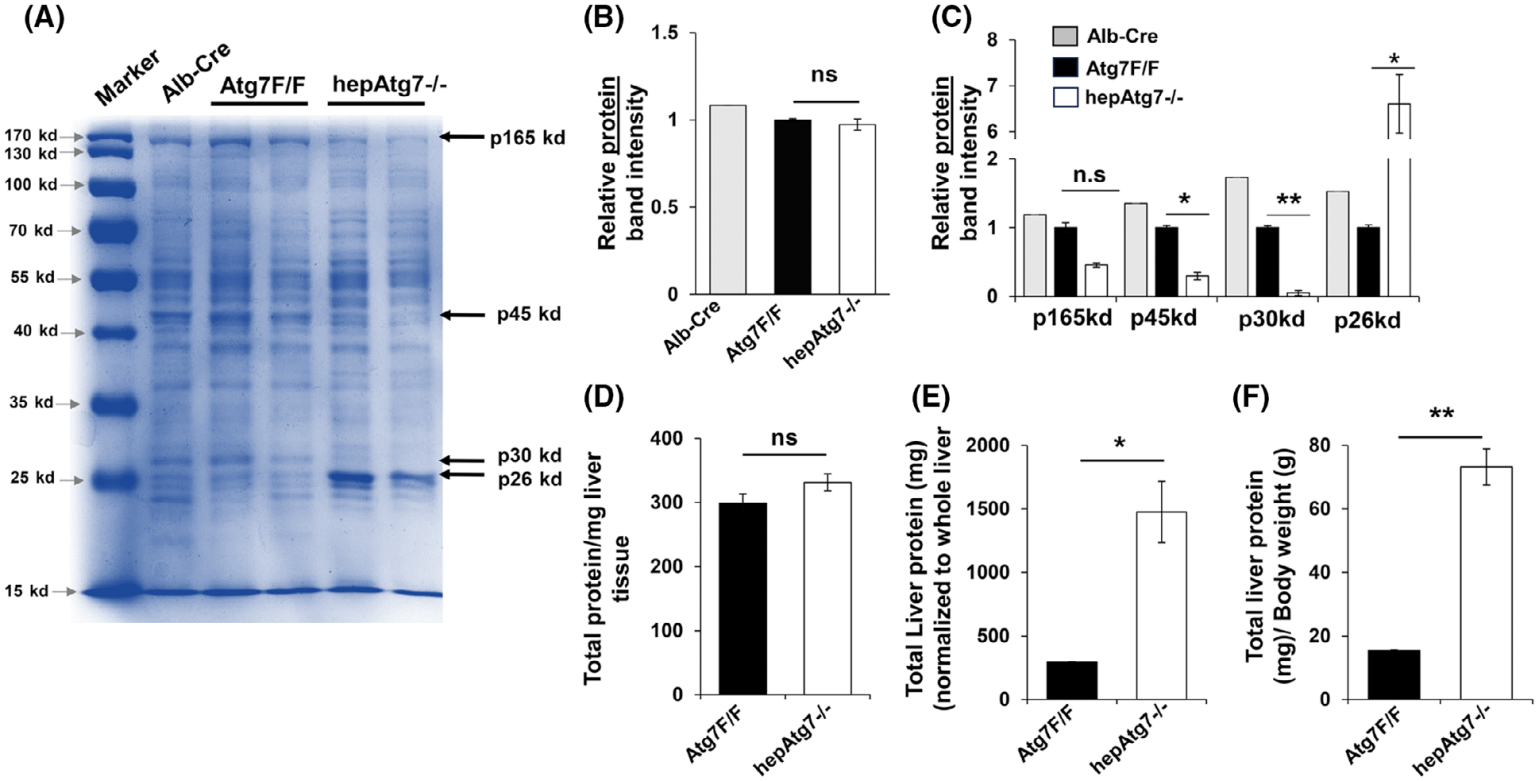
Qualitative change in hepatic proteome of *conditional* autophagy‐deficient liver. (A) Total liver lysates from 9‐week‐old mice were analyzed by CBB staining. (B) Protein band intensities in CBB stained gel were quantified by densitometry for total protein and for specific proteins (p) of different molecular weights: p165, p45, p30, and p26. (C) The protein band intensities were normalized to average overall band intensity of Atg7F/F group. (D) Hepatic total protein level normalized to milligram (mg) of liver tissue from Atg7F/F and hepAtg7−/− genotype. (E) Hepatic total protein level normalized to liver weight of Atg7F/F and hepAtg7−/− genotype. (F) Hepatic total protein level normalized to whole body weight of Atg7F/F and hepAtg7−/− genotype. Statistical analysis was conducted using a paired Student's *t*‐test. Number of mice (*n*) ≥ 3 per group. Data are expressed as the mean ± SEM. ns, not significant; *P < 0.05, **P < 0.01, ***P < 0.001.

Notably, CBB staining (Fig. 2A–C) and total hepatic protein estimation by BCA assay did not show significant differences in overall level of liver proteins (Fig. 2D) between normal and autophagy‐deficient liver. Normalization of total protein content to liver weight (LW) and body weight (BW), however, showed significant increase in total hepatic protein (Fig. 2E,F). This may be due to the massive hepatomegaly of autophagy‐deficient liver [8]. Autophagy deficiency causes massive hepatomegaly due to increased cell proliferation, hypertrophy, and infiltration of non‐parenchymal inflammatory cells [6, 8, 9, 10, 11].

Interestingly, protein expression pattern in CBB stain was distinctly different for *selected* proteins in the autophagy‐deficient liver, indicating a qualitative change in the hepatic proteome (Fig. 2A–C). Especially the protein band intensity for p165kd, p45kd, p30kd, and p26kd were noted to have differential expression patterns in autophagy‐deficient liver (Fig. 2A) when compared to normal liver. The relative band intensities for p165kd, p45kd, and p30kd were significantly decreased whereas p26kd was significantly elevated in autophagy‐deficient liver (Fig. 2C). The qualitative alteration of specific hepatic proteins—p165kd, p45kd, p30kd, and p26kd—were also observed in Atg5‐deficient liver (Fig. S2A–C). Atg5 is a another key autophagy‐related protein and an integral part of the Atg5‐Atg12‐Atg16L1 complex that catalyzes the Atg8 (LC3) lipidation essential for autophagosome formation and expansion [12]. These observations in Atg7‐and Atg5‐deficinet liver suggest that autophagy can have a direct impact on the level of selective hepatic proteins.

### Hepatic proteome is qualitatively altered in an in *inducible* model of autophagy‐deficient liver

**Fig. 3:**
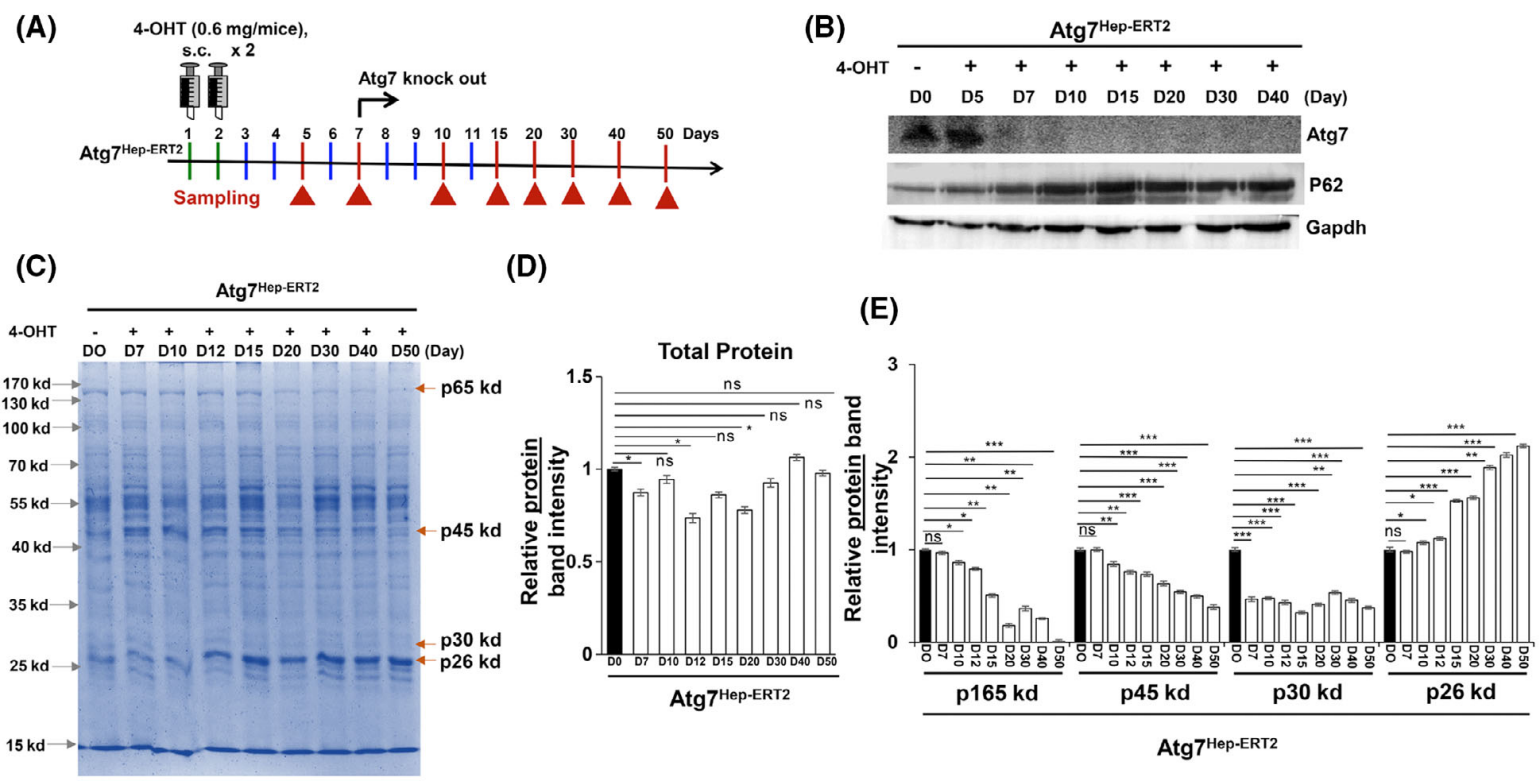
Qualitative change in hepatic proteome of *inducible model of* autophagy‐deficient liver. (A) study design of hepatic proteome analysis in an inducible autophagy‐deficient liver. Atg7^Hep‐ERT2^ mice were injected with two doses of tamoxifen (4‐OHT, 0.6 mg/mouse) subcutaneously, and liver were harvested at different days, day 0 to day 40 post 4‐OHT injection. (B) Immunoblot analysis for Atg7, P62, and Gapdh in total liver lysate prepared from Atg7^Hep‐ERT2^ mice. (C) CBB stained gel for examination of hepatic proteome in Atg7^Hep‐ERT2^ mice. (D) Quantification of overall band intensity of total proteins detected by CBB stain. The relative band intensity was normalized to the band intensities for D0 sample. (E) Densitometric quantification of specific liver protein bands p165kd, p45kd, p30kd, and p26kd detected by CBB stain. The specific band intensities were normalized to the band intensity for respective protein in D0 sample. Statistical analysis was conducted using a paired Student's *t*‐test. Number of mice (*n*) ≥ 3 per group. Data are expressed as the mean ± SEM. ns, not significant; *P < 0.05, **P < 0.01, ***P < 0.001.

To further examine and validate the specific alteration of the hepatic proteins, we next examined hepatic proteome of an *inducible* model of autophagy‐deficient mice (Atg7^Hep‐ERT2^) (Fig. S3). The inducible model of autophagy‐deficient liver was generated by crossing the Atg7 F/F mice with the AlbCre‐ERT2 mice. Atg7^Hep‐ERT2^ mice are born as wild‐type control mice with normal expression of functional Atg7 and intact autophagy process. Liver‐specific *Atg7* deletion in adult mice was induced by subcutaneously injecting tamoxifen (4‐OHT) into Atg7^HepERT2^ mice (Fig. 3A). Livers were harvested at different timepoints (day (D)0, D7, D10, D15, D20, D30, and D40) after 4‐OHT injections. Atg7 and P62 immunoblot analysis shows that Atg7 deletion was observed after D7 post 4‐OHT injection. P62, an established autophagic substrate was accumulated starting from day 7 and continued to later time points (Fig. 3B). Examination of hepatic proteome by CBB stain did not show significant differences in overall total protein band intensities for hepatic proteome between D0 and D7–D40 mice (Fig. 3C,D), similar as with the conditional autophagy‐deficient liver (Fig. 2). Notably, the relative band intensities of p165kd, p45kd, and p30kd were lower, while p26kd was higher, at different time points post Atg7 deletion (Fig. 3E). This suggests that the qualitative alteration in hepatic proteins occurs post Atg7 deletion in an inducible model of autophagy deficiency.

### Tumor bearing older autophagy‐deficient liver show similar qualitative alteration of hepatic proteome

**Fig. 4:**
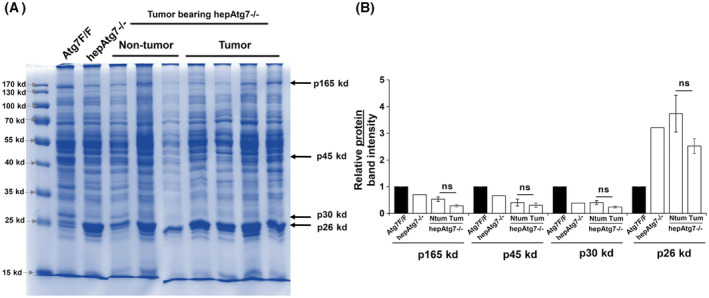
Qualitative alteration of hepatic proteome is retained in tumor bearing older autophagy‐deficient mice. (A) CBB stained gel for examination of hepatic proteome in older Atg7F/F, hepAtg7−/−(non‐tumor: Ntum) and tumor bearing hepAtg7−/− (Tumor: Tum) mice. (B) Densitometric quantification of specific liver protein bands p165kd, p45kd, p30kd, and p26kd detected by CBB stain. The specific band intensities were normalized to the band intensity for respective protein in Atg7F/F lane. Statistical analysis was conducted using a paired Student's *t*‐test. Number of mice (*n*) ≥ 3 per group. Data are expressed as the mean ± SEM. ns, not significant; *P < 0.05, **P < 0.01, ***P < 0.001.

Hepatic tumors develop spontaneously in autophagy‐deficient liver at the later stages [10, 11, 13, 14]. To determine whether the specific alteration of hepatic proteins for p165kd, p45kd, p30kd, and p26kd proteins occurs in older autophagy‐deficient liver with or without tumor, we examined the hepatic proteome in older mice of 9‐month age when they start to develop noticeable hepatic tumors. Tumor (Tum) and non‐tumor (NTum) tissue was harvested from 9 months old Atg7 deficient mice to analyze the proteomic profile. Notably, CBB staining showed similar selective alteration of specific hepatic protein in the old tumor boring autophagy‐deficient mice (Fig. 4A,B). Importantly the relative band intensities of the p165kd, p45kd, p30kd, and p26kd proteins for tumor and non‐tumor tissues remained same.

Compared to normal liver, the relative band intensities of p165kd, p45kd, and p30kd were lower while p26kd was higher in autophagy‐deficient hepatic tumor. These data correlate with our observation with the young conditional and inducible autophagy‐deficient mice. Hence, the expression of specific hepatic proteins, such as p165kd, p45kd, p30kd, and p26kd, are altered in younger and older autophagy‐deficient mice.

### DDC hepatotoxin‐containing diet cause similar qualitative alteration in hepatic proteome

**Fig. 5:**
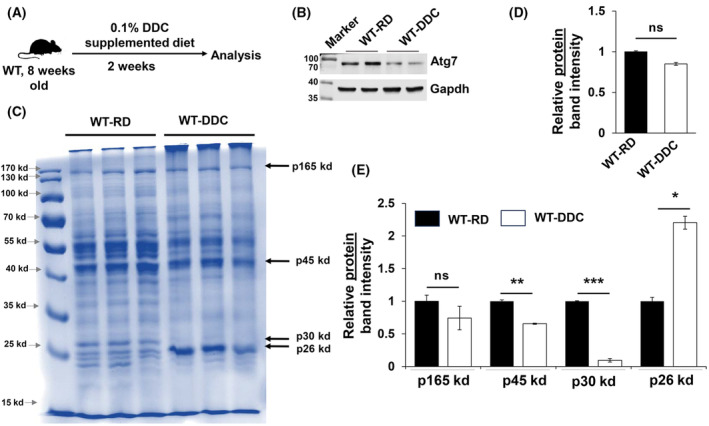
Qualitative alteration of hepatic proteome in DDC hepatotoxin model of autophagy impairment. (A) study design of hepatic proteome analysis in DDC hepatotoxin model of autophagy impairment. Eight‐week‐old Wild‐type (WT) mice were fed with regular diet (RD) and 0.1% DDC diet for 2 weeks and liver was harvested for analysis. (B) Immunoblot analysis for Atg7 and Gapdh in total liver lysate prepared from WT mice fed with RD and 2‐week DDC diet. (C) CBB stained gel for examination of hepatic proteome in WT mice fed with regular diet and 2‐week DDC diet. (D) Quantification of overall band intensity of total proteins detected by CBB stain. The overall band intensity was normalized to the band intensities for RD fed WT mice. (E) Densitometric quantification of specific liver protein bands p165kd, p45kd, p30kd and p26kd detected by CBB stain. The specific band intensities were normalized to the band intensity for respective protein in RD fed WT mice. Statistical analysis was conducted using a paired Student's *t*‐test. Statistical analysis was conducted using a paired Student's *t*‐test. Data are expressed as the mean ± SEM. ns, not significant; *P < 0.05, **P < 0.01, ***P < 0.001 (*n* = 3).

Hepatic exposure of xenobiotics can impair autophagy function [15]. Acute feeding of DDC hepatotoxin‐containing diet reduce Atg7 expression (Fig. 5A,B) and impair autophagy function [15]. Acute feeding of DDC is well known to cause accumulation of intrahepatic protein aggregates called Mallory Denk Bodies [16]. However, the status of hepatic proteome under acute exposure to DDC has not been examined. DDC diet was acutely fed to wild‐type mice for 2 weeks to assess the impact of hepatic toxin on the liver proteome (Fig. 5A). As in autophagy‐deficient liver, CBB did not demonstrate significant differences in overall total protein band intensity (Fig. 5C,D). Consistently, selective proteins—p165kd, p45kd, p30kd, and p26kd—were remarkably different in the DDC‐exposed liver, also indicating a qualitative change in the hepatic proteome (Fig. 5C). The relative band intensities of p165kd, p45kd, and p30kd were significantly lower while p26kd demonstrated a significant increase (Fig. 5E) in DDC diet exposed liver. Thus, acute DDC causes a qualitative change in the hepatic proteome likely due to autophagy functional impairment.

### Identification of autophagy‐associated altered hepatic proteins by MS

Impairment of hepatic autophagy function by genetic deletion of Atg7, Atg5 or by hepatotoxin altered the expression of selective hepatic proteins—p165kd, p45kd, p30kd, and p26kd. Next, we identified the p165kd, p45kd, p30kd, and p26kd proteins by liquid chromatography tandem mass spectrometry (LC/MS) in 9‐week‐old Atg7 group of mice. The altered protein bands (p165kd, p45kd, p30kd, and p26kd) were excised and then digested with trypsin prior to the MS analysis. The tandem mass spectra were searched against the human database. Multiple proteins were identified by MS which are enlisted in the Table S1 (p165kd), Table S2 (p45kd), Table S3 (p30kd), and Table S4 (p26kd). Out of top 20 protein list, we selected the protein with highest number of *Unique peptide* and *highest peptide spectrum match (PSM)* from the list of protein identified by the MS. MS data showed that Carbamoyl phosphate synthetase I (Cps1), Adenosyl homocysteinase (Ahcy), Carbonic anhydrase 3 (Ca3), and Glutathione *S*‐Transferase Mu 1 (Gstm1) have the highest number of unique peptides and the highest PSM values (Tables [Link], [Link], [Link], [Link]). More importantly, these proteins matched with p165kd, p45kd, p30kd, and p26kd protein band sizes respectively. Cps1 is a ligase enzyme located in the mitochondria and involved in the production of urea. Cps1 transfers an ammonia molecule to a molecule of bicarbonate that has been phosphorylated by a molecule of ATP. Ahcy is the only enzyme that mediates the reversible catalysis of *S*‐adenosylhomocysteine (SAH) to adenosine and l‐homocysteine. Endogenous Ahcy is located in cytoplasm and the nucleus. Ca3 is a mitochondrial metalloenzymes that catalyze the reversible hydration of carbon dioxide. Gstm1 is a cytosolic enzyme that functions in the detoxification of electrophilic compounds including drugs and toxin, by conjugation with glutathione.

### Alteration of specific hepatic protein in autophagy‐deficient liver relates to transcriptional regulation but not due to impaired lysosomal degradation

**Fig. 6:**
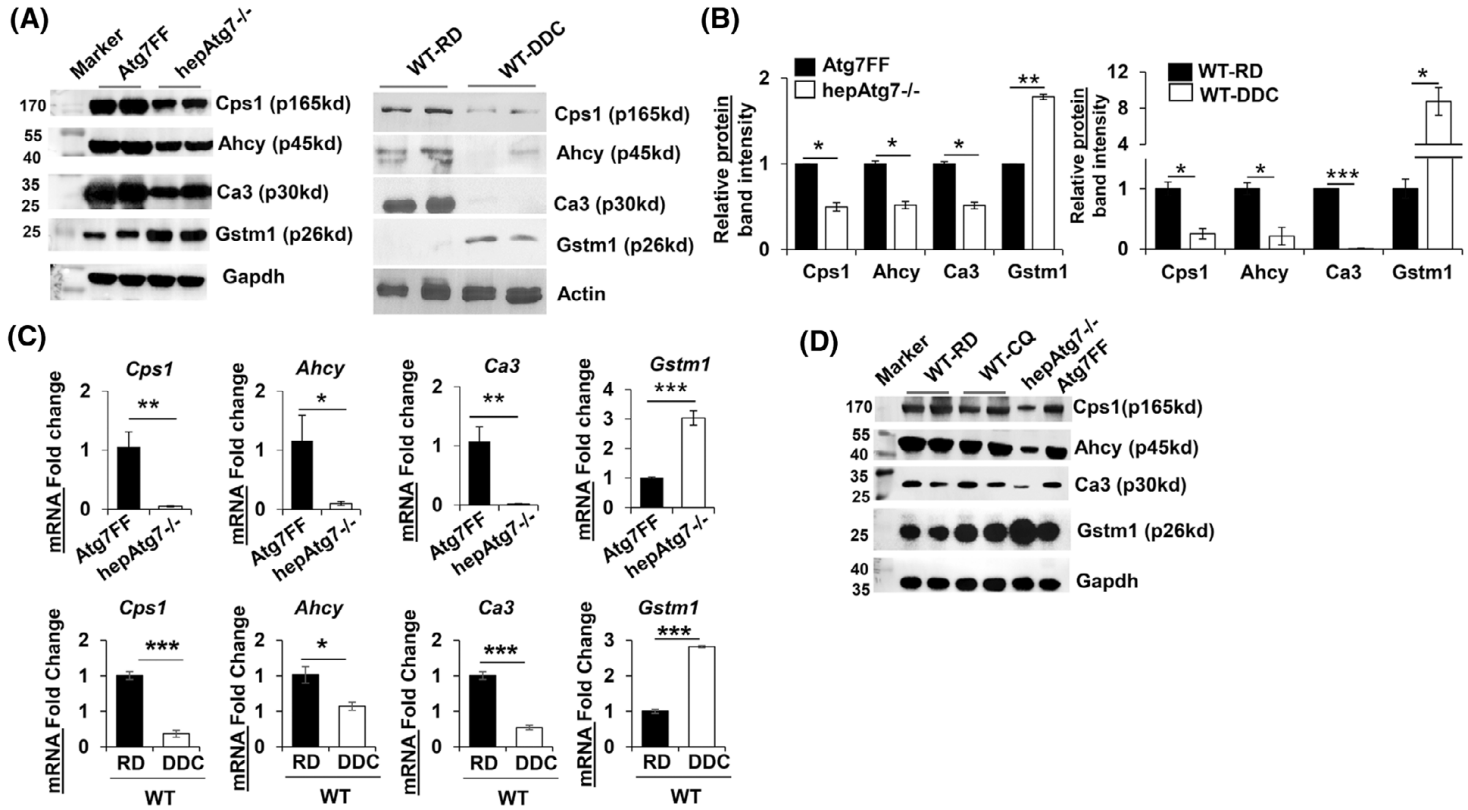
Qualitative alteration of hepatic proteome in autophagy‐impaired liver is related to transcriptional alteration. (A) Liver lysates from 9‐week‐old Atg7F/F and hepAtg7−/− mice or wild‐type mice fed with regular diet (RD) or DDC diet for 2 weeks were analyzed by immunoblotting for Cps1, Ahcy, Ca3, Gstm1, and Gapdh. (B) Protein band intensities in immunoblots were quantified by densitometry and normalized to Gapdh. (C) Quantitative PCR analysis for Cps1, Ahcy, Ca3, and Gstm1 mRNA expression in the indicated genotypes. The mRNA expression levels were normalized to actin. (D) Liver lysates from 9‐week‐old WT mice treated with or without Chloroquine (CQ) were analyzed by immunoblotting for Cps1, Ahcy, Ca3, Gstm1 and Gapdh. CQ (30 mgkg^−1^ body weight) was injected intraperitoneally once a day for six consecutive days. Liver samples were harvested on the seventh day for immunoblot analysis. Statistical analysis was conducted using a paired Student's *t*‐test. Data are expressed as the mean ± SEM. ns, not significant; *P < 0.05, **P < 0.01, ***P < 0.001 (*n* = 2 for western blot analysis and *n* = 3 for qPCR analysis).

Next, we validated the MS data of the altered hepatic proteins (p165kd, p45kd, p30kd, and p26kd) by western blot analyses. Liver lysates from 9‐week‐old Atg7F/F and hepAtg7−/− mice were analyzed for Cps1, Ahcy, Ca3, and Gstm1 protein expressions by western blot as these proteins showed the highest number of *Unique peptide* and *highest peptide spectrum match (PSM)* (Tables [Link], [Link], [Link], [Link]). Western blot analysis showed that the protein expression levels of Cps1, Ahcy, Ca3 were significantly decreased, while Gstm1 (p26kd) protein level was significantly elevated (Fig. 6A,B) in autophagy‐deficient liver. Similar expression levels of these proteins were observed in mice fed with a DDC diet and exhibiting impaired autophagy function (Fig. 6A,B). This suggests that the corresponding p165kd, p45kd, p30kd, and p26kd protein bands identified in CBB stain related to Cps1, Ahcy, Ca3, and Gstm1 protein respectively. This observation also further validates the proteomic alteration observed in autophagy‐deficient liver.

Altered proteomic expression in autophagy‐deficient liver could be related to their change in mRNA expression level or due to impaired protein degradation via lysosome. We first examined the possible role of mRNA expression in the alteration of these hepatic proteins. Indeed, examination of mRNA expression of *Cps1*, *Ahcy*, and *Ca3* showed significant downregulation whereas *Gstm1* was remarkably upregulated (Fig. 6C) in autophagy‐deficient liver. Similar expression levels of these mRNAs were observed in mice fed with a DDC diet and exhibiting impaired autophagy function (Fig. 6C). This suggests that altered hepatic proteome in autophagy‐deficient liver is likely due to alteration in the transcript expression level but not related to the impairment in the autophagic lysosomal degradation.

To rule out the role of impaired autophagic lysosomal degradation in the alteration of these specific hepatic proteins, we examine the protein expression level of Cps1, Ahcy, Ca3, and Gstm1 in liver sample of wild‐type mice chronically treated with chloroquine (CQ) for 6 consecutive days. Notably, immunoblot examination did not show any remarkable changes in the hepatic protein expression level of Cps1, Ahcy, Ca3, and Gstm1 between vehicle and CQ‐treated wild‐type mice (Fig. 6D). Thus, these data suggest that alteration of selective hepatic protein expression of Cps1, Ahcy, Ca3, and Gstm1 in autophagy‐deficient mice is not due to impaired autophagic lysosomal degradation. The alteration of selective hepatic protein expression of Cps1, Ahcy, Ca3, and Gstm1 is due to transcriptional changes that could be related to autophagy deficiency.

### Nuclear factor erytheroid‐derived‐2‐like 2 (Nrf2) mediates transcriptional expression of specific hepatic proteins in autophagy‐deficient liver

Autophagy deficiency persistently activates Nrf2 transcription factor [17, 18]. Under normal conditions, Nrf2 is bound to the E3 ubiquitin ligase adaptor protein, Kelch‐like ECH‐associated protein 1 (Keap1), which leads to its ubiquitylation and proteasomal degradation [18]. When autophagic function is compromised and autophagic substrate protein, P62/SQSTM1 accumulates, Keap1 is sequestered by P62 and can no longer bind Nrf2, leading to increased Nrf2 signaling. Nrf2 binds to the antioxidant response element (ARE) present in the promoter region of the target gene to induce its expression [19, 20].

**Fig. 7:**
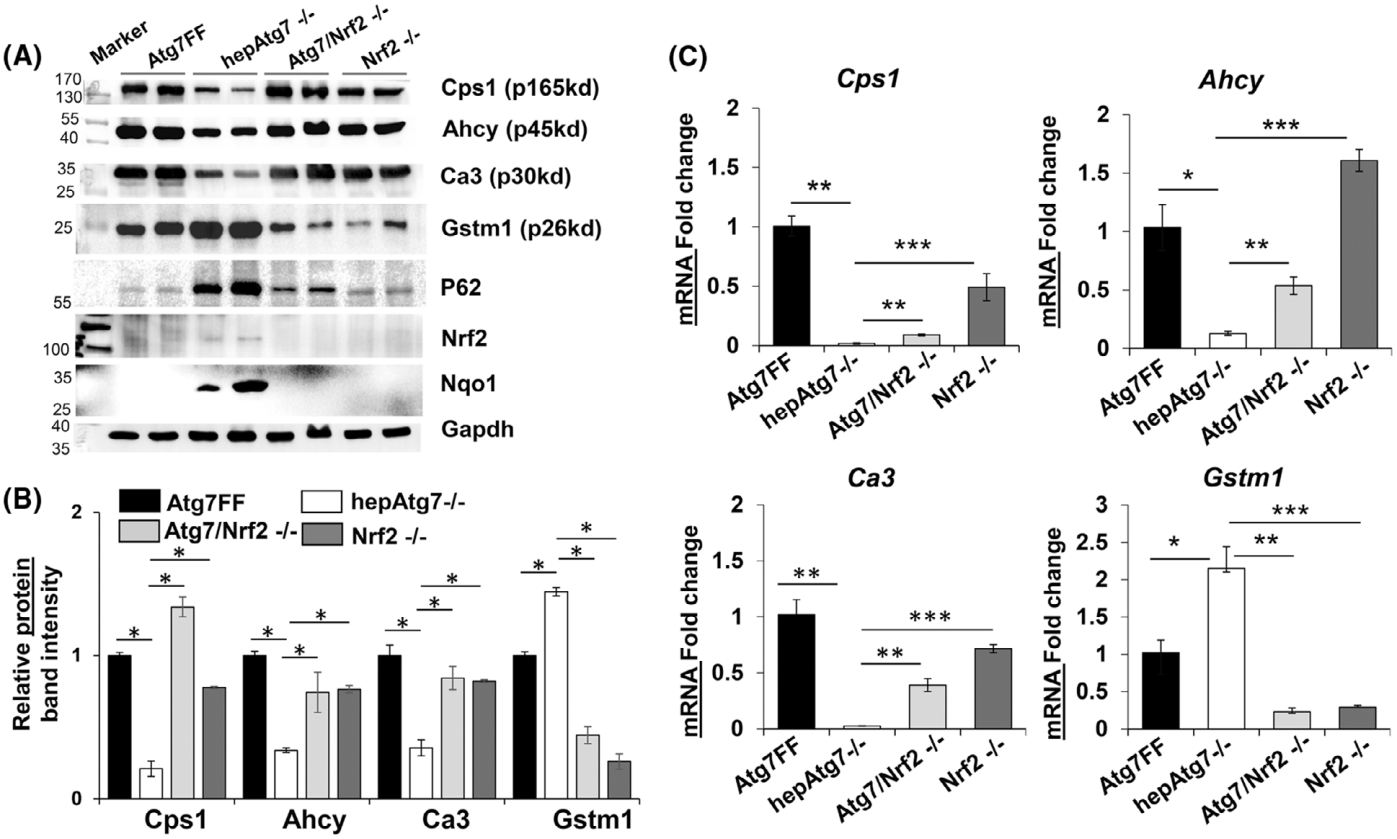
Nrf2 mediated transcriptional alteration influence the hepatic proteome. (A) Liver lysates from 9‐week‐old Atg7F/F, hepAtg7−/−, Atg7/Nrf2−/− and Nrf2−/− mice were analyzed by immunoblotting for Cps1, Ahcy, Ca3, Gstm1, Nrf2, Nqo1, and Gapdh. (B) Protein band intensities in immunoblots were quantified by densitometry and normalized to Gapdh. (C) Quantitative PCR analysis for Cps1, Ahcy, Ca3, and Gstm1 mRNA expression. The mRNA expression levels were normalized to actin. Statistical analysis was conducted using a paired Student's *t*‐test. Number of mice (*n*) ≥ 3 per group. Data are expressed as the mean ± SEM. ns, not significant; *P < 0.05, **P < 0.01, ***P < 0.001.

Since altered hepatic protein is related to transcriptional alteration and autophagy deficiency‐mediated Nrf2 activation could play role in the altered hepatic proteome. We next sought to understand the molecular mechanism regulating the proteomic alteration in autophagy‐deficient liver. For this study, we utilized Atg7/Nrf2−/− double knock out and Nrf2−/− mice. We examined the protein and mRNA expression level of the Cps1, Ahcy, Ca3, and Gstm1 proteins in the liver of 9‐week‐old Atg7F/F, hepAtg7−/−, Atg7/Nrf2−/− and Nrf2−/− mice. Knocking out the Nrf2 in autophagy‐deficient mice reversed the protein and mRNA expression of the altered hepatic proteins back to normal level (Fig. 7A–C). These observations suggest that persistent activation of NRF2 due to autophagy impairment leads to the altered hepatic proteome in autophagy‐deficient liver.

## Discussion

Autophagy plays a critical role in regulating the hepatic proteome. It is generally assumed that autophagic degradation would modulate the level of the hepatic proteome. Supporting this notion, many hepatic proteins such as P62/SQSTM1, NcoR1, Cry1, Yap, etc. [6, 8, 21, 22] have been reported to be selective targets of autophagic degradation. Hence, impaired autophagy leads to their hepatic accumulation. However, this study indicates that besides the accumulation of the hepatic proteome due to impaired autophagic clearance, there is a downregulation of specific hepatic proteins, implying a differential regulation of the hepatic proteome by autophagy. This concept is demonstrated by the selective downregulation in the levels of hepatic proteins such as Cps1, Ahcy, and Ca3 (Figs 1 and 6) as shown in Figs 1 and 6, in livers deficient in autophagy.

**Fig. 8:**
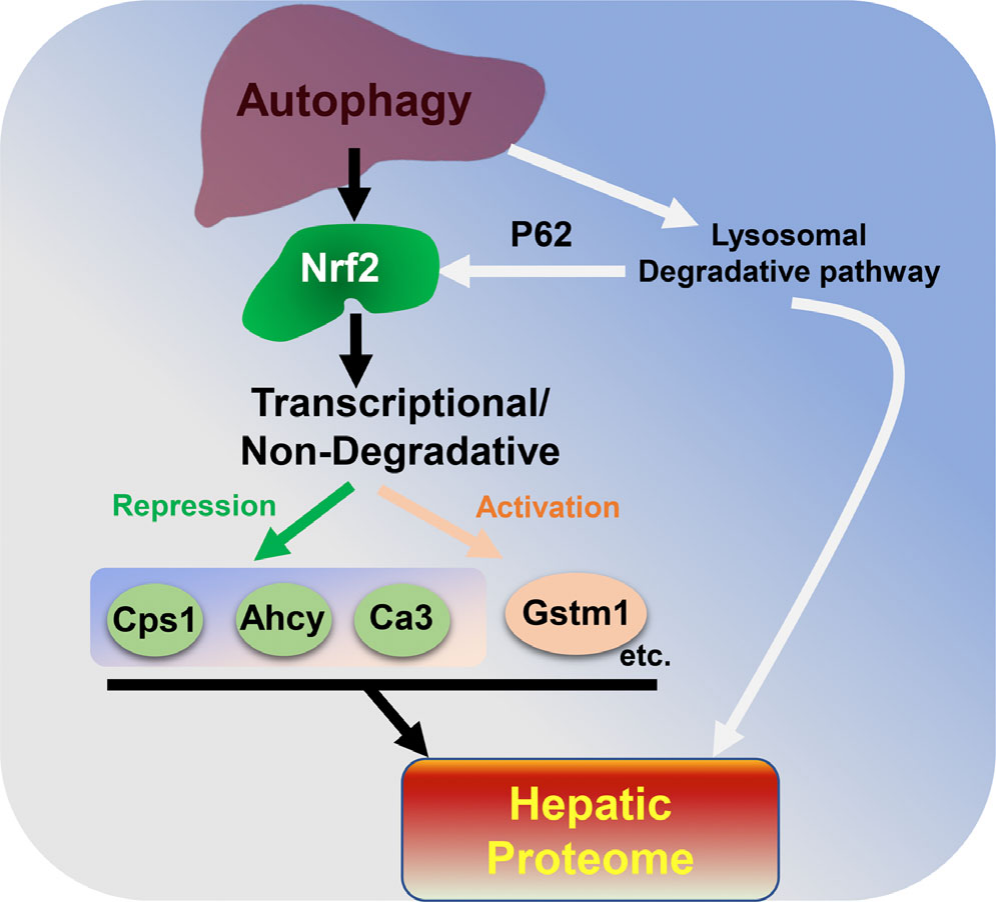
Schematic model showing how autophagy regulates hepatic proteome. Autophagy deficiency can affect the hepatic proteome through two primary pathways: (a) the lysosomal degradative pathway, and (b) the transcriptional non‐degradative pathway. A deficiency in autophagy leads to continuous activation of Nrf2 due to the accumulation of P62. This, in turn, can transcriptionally activate or repress downstream target genes, thereby influencing the expression levels of various hepatic proteins (such as Cps1, Ahcy, Ca3, and Gstm1) and ultimately affecting the overall hepatic proteome.

It is possible that other proteins may also show similar changes since CBB stain may not be as sensitive to detect them. These selective hepatic proteins are altered not by impaired autophagic lysosomal degradation, but rather by a non‐degradative transcriptional process (Figs 6C and 7C). Hence, this study provides a new mechanistic layer for the autophagic regulation of the hepatic proteome via transcriptional non‐degradative process (Fig. 8).

When autophagy is impaired, P62 accumulates, which competes with Keap1 for binding to Nrf2, leading to persistent accumulation of Nrf2 [17]. Nrf2 can also transcriptionally upregulate P62 expression [23], resulting in a vicious cycle of P62 accumulation and Nrf2 activation. Nrf2 is an anti‐oxidative stress‐related transcription factor that binds to specific regions called anti‐oxidative response elements (ARE) of target genes promoter [19, 24]. We have previously shown that Nrf2 can bind to the ARE element in the promoter of caspase‐11 and transcriptionally upregulate its expression for inflammasome activation [25]. On the other hand, our another study shows that Nrf2 can transcriptionally suppress FXR nuclear receptors and impact the hepatic bile acid metabolism [26]. Nrf2 may also transcriptionally regulate Cps1, Ahcy, Ca3, and Gstm1 expression levels similarly by binding to the ARE element present in their promoters. Nrf2 might collaborate with transcriptional repressors like NcoR1, which accumulates in conditions [22] where autophagy is impaired, potentially playing a role in the transcriptional suppression of certain genes through FXR. Our preliminary analyses by JASPER and MatInspector show the presence of ARE elements in the promoter regions of Cps1, Ahcy, Ca3, and Gstm1 (data not shown). However, Cps1, Ahcy, and Ca3 expression is severely suppressed whereas Gstm1 expression is elevated in the autophagy‐deficient liver. How a single transcription factor such as Nrf2 could simultaneously suppress (Cps1, Ahcy, and Ca3) and activate (Gstm1) the gene expression by binding to the ARE element is unknown. There is a possibility that Nrf2 can associate with different binding partners such as NcoR1 or MafG to differentially express genes. Nrf2 activates transcription by interacting with small Maf proteins, such as MafG [19]. This is the case for Nrf2‐mediated regulation of Gstm1 expression [24] as its expression is upregulated in the autophagy‐deficient liver. It is possible, however, that Nrf2 interacts with a NcoR1 or a new protein factor to bind to the ARE element of the promoter and repress the expression of Cps1, Ahcy, and Ca3. Nrf2 has been reported to bind to replication protein A 1 (RPA1) instead of MafG to cause ARE‐dependent gene repression [27]. RPA1 competes with MafG for Nrf2 binding, resulting in Nrf2‐RPA1 interacting with the ARE region and enhancing promoter activity [27]. Whether NcoR1 or RPA1 plays a Nrf2 gene repressive function in autophagy‐deficient livers is unknown. It is perplexing how the Nrf2 pool would interact with MafG (to activate) and NcoR1 or RPA1 (to repress) in the same system (autophagy‐deficient liver) to simultaneously activate and repress the genes. Alternatively, the presence of specific repressor protein/s that can interact with the Nrf2‐MafG complex at the respective promoters of Cps1, Ahcy, Ca3, and Gstm1 would determine the simultaneous activation or repression of these genes. Future mechanistic studies will attempt to identify Nrf2‐mediated gene repressive function.

## Methods

### Animal models

Wild‐type (C57BL/6) mice, Atg7 floxed mice (Atg7F/F), Atg5 floxed mice (Atg5F/F), Atg7‐deficient mice (hepAtg7−/−), Atg5‐deficient mice (hepAtg5−/−), Atg7/Nrf2−/− and Nrf2−/− were used in this study. Atg7F/F (RBRC02759) mice and Atg5F/F (RBC02975) were purchased from the RIKEN BioResource Research Center [9, 28]. Nrf2−/− mice were obtained from Jackson Laboratory, Bar Harbor, ME, USA (B6.129X1‐*Nfe2l2tm1Ywk*/J, stock no. 017009). Atg7F/F or Atg5F/F mice were crossed with albumin‐Cre recombinase (Alb‐Cre) mice (C57BL/6) (Jackson Laboratory) to generate hepAtg7−/− and hepAtg5−/−mice respectively. The liver‐specific inducible Atg7‐deficient mice (Atg7^Hep‐ERT2^) were generated by crossing Atg7F/F mice with AlbCre‐ERT2 mice. Deletion of Atg7 was induced by subcutaneous administration of tamoxifen (6 mg·day^−1^, ×2 days). Atg7F/F and Alb‐Cre mice were further crossed with Nrf2−/− (Jackson Laboratory) to generate Atg7/Nrf2−/− mice as previously described [26, 29]. Mice were housed in a pathogen‐free facility at Tulane University School of Medicine. Mice were maintained on a 12‐h light/12‐h dark cycle with free access to food and water. A mixture of male and female mice aged between 8 and 12 weeks or older 9 and 12 months were used in this study. Mice ages are indicated in the figure legends. This preclinical study adhered to Animal Research: Reporting of the *In Vivo* Experiments (ARRIVE guideline 2.0). All animal procedures were approved and performed in accordance with guidelines and regulations of the Institute Animal Care and Use Committee (IACUC) of Tulane University (IACUC approval number 1809).

### DDC (3,5‐diethoxycarbonyl‐1,4‐dihydrocollidine) hepatotoxin diet

Adult wild‐type mice of the same age (8 weeks, approximately 25 gram) were randomly assigned to either the regular diet group or the DDC diet group. Those in the regular diet group were fed a regular chow diet of commercially available mouse food pellets (free of xenobiotics). Mice in the DDC diet were fed a diet containing 0.01% of DDC for 2 weeks. The mice were then humanely sacrificed, and their livers were collected and frozen to be used for downstream experiments.

### Immunofluorescence staining

A portion of the mice liver tissues were separately fixed with 4% paraformaldehyde and sucrose. Frozen blocks were prepared with Scigen Tissue‐Plus optimum cutting temperature compound (Thermo Fischer Scientific, Waltham, MA, USA). Frozen sections (4 μm) were prepared from frozen blocks and placed on unstained slides where they were then subjected to antigen retrieval using citrate buffer (pH 6.0). Frozen sections were subjected to antigen retrieval using citrate buffer (pH 6.0) after deparaffinization. Slides were permeabilized and blocked with 10% goat serum in PBS containing 0.5% Triton X (PBS‐Tx) and glycine for 1 h and then incubated overnight at 4 °C with primary antibody (Table S5) diluted in PBS. Sections were washed in PBS‐Tx, followed by incubation with fluorochrome‐conjugated secondary antibodies. Hoechst 33342 (1 μg/mL) was used for staining nuclei. Images were obtained using an Olympus IX73 inverted microscope with an Olympus DP80 color digital camera and the Olympus cellSens software.

### Coomassie brilliant blue (CBB) staining

Total liver protein lysate was prepared from the liver samples using RIPA lysis buffer containing protease inhibitor cocktail. The liver lysate was centrifuged after homogenizing the samples using an electric homogenizer. Lysates were estimated for total protein using a BCA assay (using commercially available kit following standard protocol) and then separated by sodium dodecyl sulfate polyacrylamide gel electrophoresis (SDS/PAGE). Gel was washed three times to remove extra SDS and incubated in CBB for 1 h at room temperature. Gels were re‐washed with deionized water and imaged using Bio‐Rad Gel imager, Hercules, CA, USA.

### Western blot analysis

Total liver protein lysate was separated by SDS/PAGE. After SDS/PAGE, the proteins were transferred to 0.4‐micron PVDF membrane. The membranes were blocked in TBS with 0.1% Tween 20 (TBS‐T) and 5% non‐fat dry milk powder for at least 1 h. The membranes were then incubated with primary antibodies (Table S1) overnight at 4 °C. The membranes were washed with TBS‐T and incubated with a horseradish peroxidase‐conjugated secondary antibody for 1 h. Blots were visualized using the Immunobilion chemiluminescence system (Burlington, Millipore, MA, USA) kit and Bio‐Rad chemiluminescence machine scanner. The densitometry analysis of immunoblot images was performed using quantity one software (Bio‐Rad). Densitometry values were normalized to the loading control (GAPDH) and then converted to units relative to the untreated control.

### Quantitative PCR

A GeneJET RNA Purification Kit (Thermo Fisher Scientific) was used to extract the total RNA from homogenized liver samples, according to the manufacturer's protocol. One microgram total RNA was then used to synthesize cDNA using a M‐MLV Reverse Transcriptase Enzyme System (Life Technologies, Carlsbad, CA, USA; Thermo Fisher Scientific) and OligoT primers. qPCR was performed using SYBR Green Master Mixes on a Quanta studio 3 PCR machine (Life Technologies–Applied Biosystems, Waltham, MA, USA; Thermo Fisher Scientific), using gene‐specific primers included in the Table S6. Gene expression was calculated using the $2^{–\Delta \Delta C_{t}}$ method and normalized to the housekeeping gene Actin.

### Sample preparation for liquid chromatography tandem mass spectrometry (LC/MS)

Gel samples were rinsed with deionized water and then diced into smaller sizes. These gel pieces were then vigorously mixed in a destaining buffer containing 25 mm ammonium bicarbonate and 50% acetonitrile (ACN). The step was repeated until the gel bands were clear and subsequently dehydrated in 100% ACN. The gel pieces were reduced with 25 mm dithiothreitol for 1 h, followed by alkylation using 20 mm iodoacetamide in the dark at room temperature. The gel pieces were digested with trypsin (Promega, Madison, WI, USA) at 37 °C overnight. The digested peptides were extracted with 1% formic acid (FA) in H_2_O and an additional extraction was processed using 70% ACN/5% FA in water. The extracted peptides were dried using a Vacufuge (Eppendorf, Hamburg, Germany) and then reconstituted in 2% ACN/0.1% FA in water. The resulting peptide mixtures were then ready for LC–MS/MS analysis.

### LC–MS analysis

Peptide samples were analyzed using an Q Exactive HF‐X Orbitrap MS (Thermo Fisher Scientific) coupled with the UltiMate 3000 UHPLC (Thermo Fisher Scientific). Peptides were loaded onto a PepMap™ C18 trap column (5 μm, 300 μm × 5 mm; Thermo Fisher Scientific) and an Easy‐Spray™ PepMap™ C18 (2 μm, 75 μm × 50 cm; Thermo Fisher Scientific) analytical column. The mobile phases were comprised of 0.1% FA in water as solvent A and 80% ACN/0.1% FA in water as solvent B, with a flow rate at 0.35 μL·min^−1^. Peptides were eluted with 5% for 3 min, and then with a linear gradient from 5% to 23% buffer B over 85 min. Following this linear separation, the gradient was increased to 40% buffer B over 8 min and then increased to 98% buffer B over 6 min. The column was maintained at 98% buffer for 4 min and then equilibrated with 5% buffer B prior to a subsequent injection.

Data were acquired using a top 15 data‐dependent acquisition mode (DDA). The spray voltage was 1800 V in the positive ion mode, and the ion transfer tube temperature was set at 275 °C. Precursor spectra were collected from 300 to 1650 *m/z* at a resolution of 60 000 (AGC target of 3e6, max IT of 25 ms). MS/MS scans were collected from 200 to 2000 *m/z* at a resolution of 15 000 (AGC target of 1e5, max IT of 28 ms) with an isolation window of 1.4 *m/z* and a collision energy of 27.

### Data analysis

The LC–MS/MS raw data were processed using the Sequest algorithm in proteome discoverer 2.4.1.15 (Thermo Fisher Scientific) against Uniprot *mus musculus* (UP000000589) proteome database to obtain peptide and protein identifications. In all searches, trypsin was specified as the enzyme for protein cleavage, allowing up to two missed cleavages. Oxidation (M) and carbamidomethylation (C) were set as dynamic and fixed modifications, respectively. Both peptide spectrum match and protein false discovery rate (FDR) was set to 0.01 and determined using a percolator node. Relative protein quantification of the proteins was performed using the label‐free quantification workflow of proteome discoverer 2.4.1.15.

### Statistical analysis

Data are presented in figures as the mean with error bars representing ± SEM. Statistical analysis was performed using *P* values from at least two samples, calculated using a two‐tailed Student's *t*‐test for paired group comparisons *P* values of < 0.05 were considered statistically significant, denoted by “*”, while *P* values of < 0.01 were denoted by “**”, and *P* values of < 0.001 were denoted by “***”.

## Conflict of interest

The authors declare no conflict of interest.

## Author contributions

KB conducted experiments, acquired data, analyzed data, and wrote the manuscript. SJ, AL, GM, AC, AAC, KT, AM, LS conducted experiments, acquired data, and analyzed data. GL, BPB, JF designed research studies, analyzed the data. BK designed research studies, conducted experiments, acquired data, analyzed data, and wrote the manuscript.

## Supporting information


**Fig. S1.** Generation and validation of hepatocyte specific autophagy‐deficient mice.
**Fig. S2.** Qualitative change in hepatic proteome of Atg5‐deficient liver.
**Fig. S3.** Generation of hepatocyte specific inducible autophagy‐deficient mice.

**Table S1.** Mass spectrometry characterization of p165kd protein.

**Table S2.** Mass spectrometry characterization of p45kd protein.

**Table S3.** Mass spectrometry characterization of p30kd protein.

**Table S4.** Mass spectrometry characterization of p26kd protein.

**Table S5.** Antibodies list.

**Table S6.** Primers list.

## Data Availability

All experimental data generated and/or analyzed during the study are available from the Corresponding author on reasonable request.
