# Supplementary material for: Transcriptional changes impact hepatic proteome in autophagy‐impaired liver
Source: FEBS Open Bio. 2024 Sep 16;14(11):1851–63. doi: 10.1002/2211-5463.13898 (PMC11532973; doi:10.1002/2211-5463.13898)
Supplement: Supplementary file 1 — Fig. S1. Generation and validation of hepatocyte specific autophagy‐deficient mice. Fig. S2. Qualitative change in hepatic proteome of Atg5‐deficient liver. Fig. S3. Generation of hepatocyte specific inducible autophagy‐deficient mice. [file FEB4-14-1851-s002.pdf]

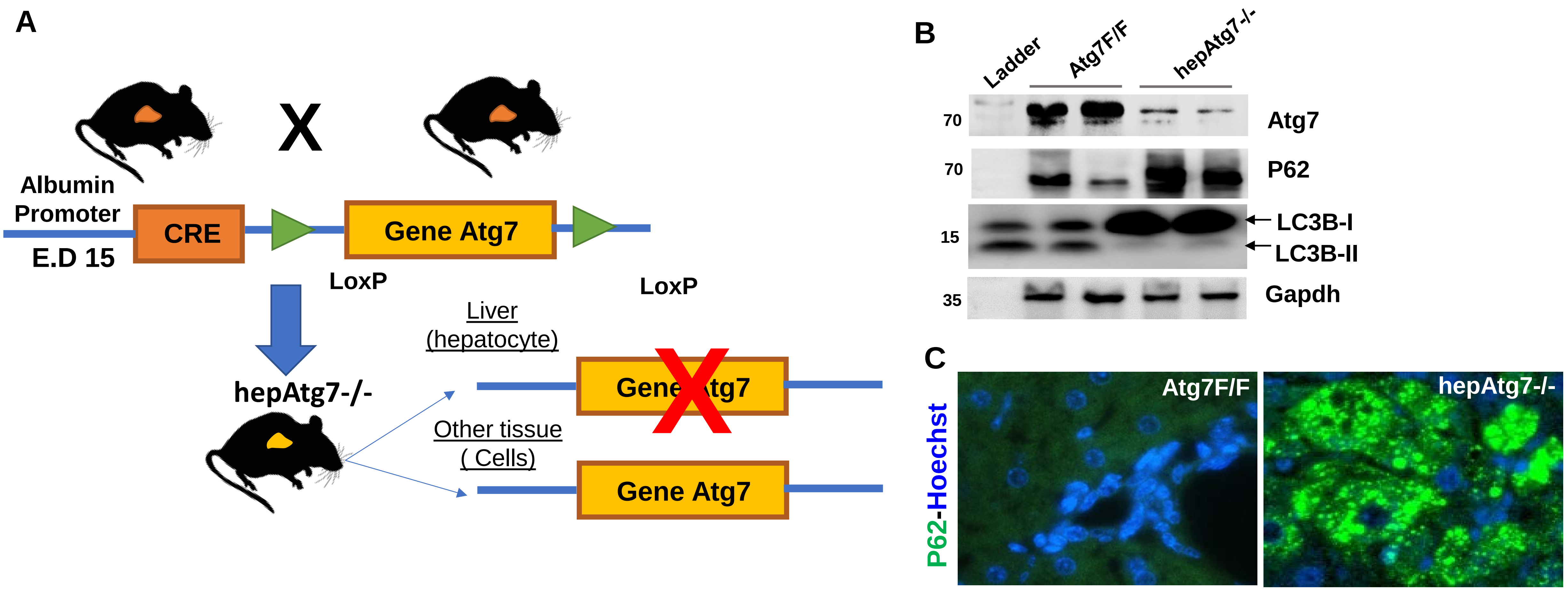

Supplementary Figure 1. Generation and validation of hepatocyte specific autophagy-deficient mice. (A) Schematic of generation of hepatocyte specific autophagy deficient (hepAtg7-/-) mouse model. Atg7 floxed mice (Atg7F/F) were crossed with Alb-Cre mice to generate Atg7-deficient mice]. (B) Immunoblot analysis for Atg7, P62, LC3B, and Gapdh in total liver lysate prepared from Atg7F/F and hepAtg7-/- genotypes. (C) P62/SQSTM1 immunofluorescence imaging to determine the accumulation of intrahepatic P62/SQSTM1 in autophagy-deficient liver.

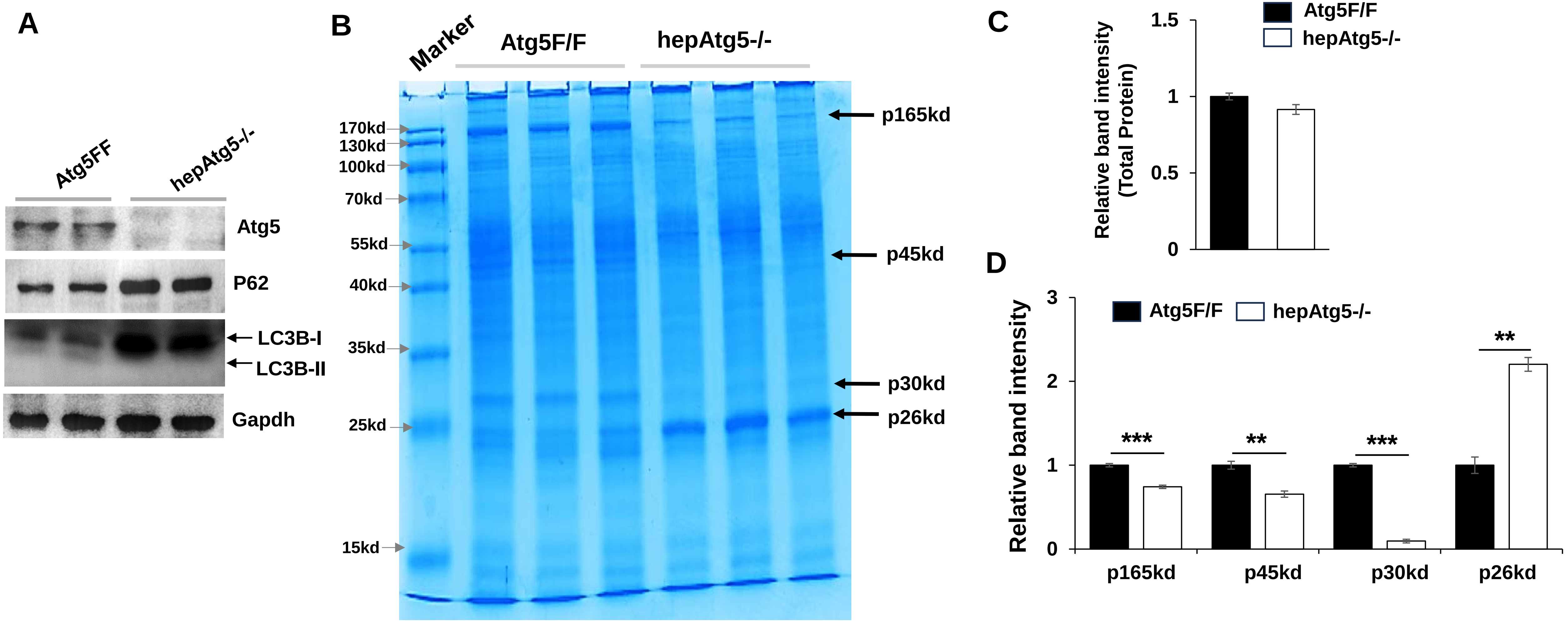

Supplementary Figure 2. Qualitative change in hepatic proteome of Atg5-deficient liver. (A) Immunoblot analysis for Atg5, P62, LC3B, and Gapdh in total liver lysate prepared from Atg5F/F and hepAtg5-/- genotypes. (B) CBB stained gel for examination of hepatic proteome in Atg5F/F and hepAtg5-/- mice. (C) Quantification of overall band intensity of total proteins detected by CBB stain. The overall band intensity was normalized to the band intensities for Atg5F/F mice. (D) Densitometric quantification of specific liver protein bands p165kd, p45kd, p30kd and p26kd detected by CBB stain. The specific band intensities were normalized to the band intensity for respective protein in Atg5F/F mice. Data are expressed as the mean ± SEM. n.s not significant $^{*}P \geq 0.05$, $^{**}P \geq 0.01$, $^{***}P \geq 0.001$.

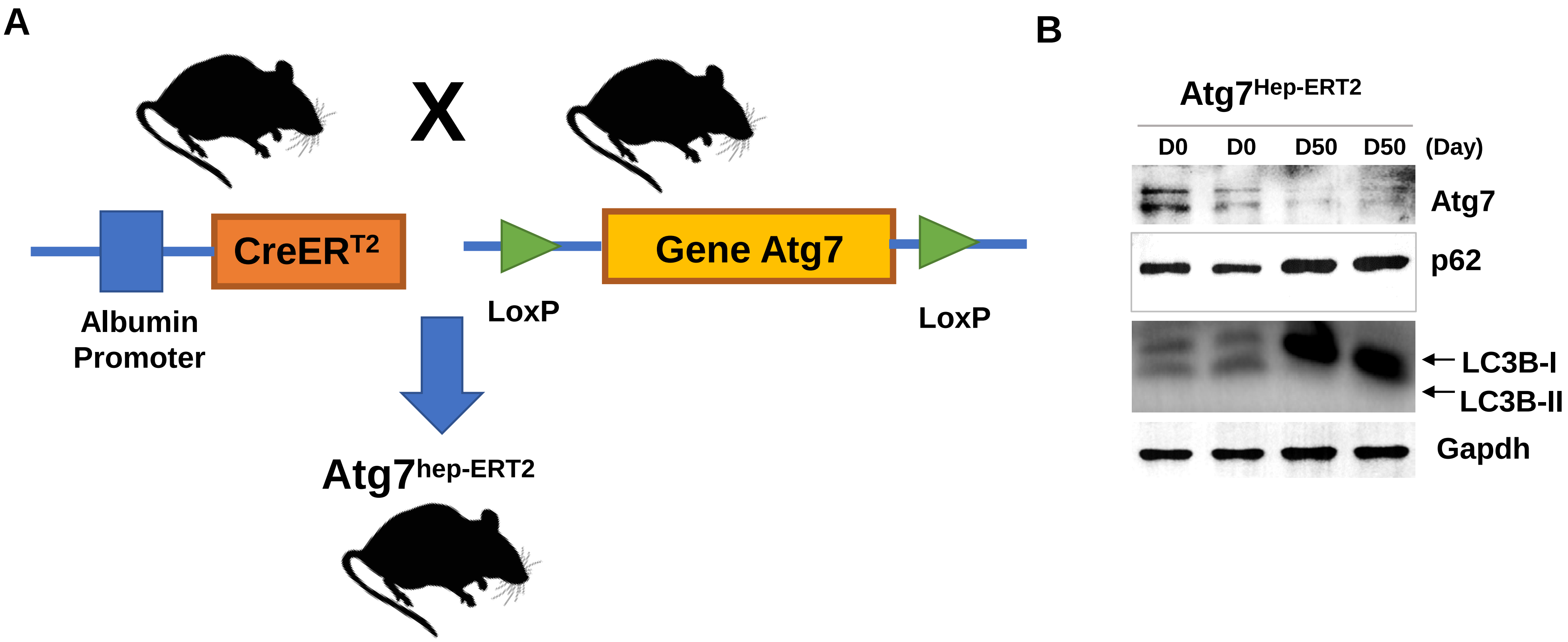

<u>Supplementary Figure 3</u>. Generation of hepatocyte specific inducible autophagy-deficient mice. (A) Schematic of generation of hepatocyte specific autophagy deficient Atg7$^{Hep\text{-}ERT2}$ mice model. Atg7 floxed mice (Atg7F/F) were crossed with Alb-ERT2-Cre mice to generate Atg7-deficient mice. (B) Immunoblot analysis for Atg7, P62, LC3B, and Gapdh in total liver lysate prepared from Atg7$^{Hep\text{-}ERT2}$ mice injected with or without tamoxifen(4-OHT, 0.6 mg/mouse) subcutaneously, and liver were harvested day 0(D0) and day 50(D50) post 4-OHT injection.
